# Fragments, Immersivity, and Reception: Punchdrunk on Aeschylus’ *Kabeiroi*

**DOI:** 10.1007/s12138-020-00578-9

**Published:** 2021-03-06

**Authors:** Emma Cole

**Affiliations:** University of Bristol, Bristol, UK

## Abstract

A preoccupation with fragmentation has defined many recent responses to antiquity. Within scholarship this focus takes the form of poststructuralist-informed readings, which highlight how any text can be perceived as fragmentary due to the epistemologies that we use to frame our readings. Within artistic practice there is a corresponding privileging of fragmentation through the dismembering of text. Yet one does not need to deconstruct a text, or to point to the gaps in meaning that persist in any textual encounter, to think through fragmentation.

In this essay, I propose that utilising actual fragments within contemporary theatre is not simply an extension in scale of wider practice but represents a qualitatively different endeavour which holds unique benefits. I suggest that fragmentary texts represent fertile material for contemporary immersive performance, as the sense of lack contained within their form provides a productive impetus for an audience's creation of the unified imaginary world necessary for a ‘deep' form of immersion. Fragments and immersive theatre make for a unique partnership as the fragmentary source text holds synergy with the form of immersive performance, where a complete or ideal experience remains an ever-elusive ambition. I make my argument through an analysis of Punchdrunk's 2017 Kabeiroi, which turned the surviving fragments of Aeschylus' Kabeiroi into a four-to-six-hour immersive experience. I argue that a sense of yearning and incompletion is inevitable in the reception of ancient fragments, but that within immersive experiences this becomes a genuinely productive force. Punchdrunk's approach, I conclude, should be considered a useful method for other artists and represents a new possible direction for classical performance reception.

A preoccupation with fragmentation has defined many recent scholarly and creative responses to antiquity. Within scholarship this focus takes the form of poststructuralist-informed readings of texts, which highlight how any text can be perceived of as fragmentary due to the epistemologies that we use to frame our readings. Within artistic practice there is a corresponding privileging of fragmentation through the dismembering of text. Eleftheria Ioannidou’s analysis of theatrical responses to Greek tragedy since the 1970s, for example, reveals how productions correspond to the poststructuralist approach to text and textuality by privileging a ‘Dionysiac’ fragmentation over ‘Apolline’ formal integrity.^1^ Erika Fischer-Lichte’s work also attests to this phenomenon when she draws upon the sacrificial terminology of a *sparagmos* and *omophageia*—a tearing apart and consumption of raw flesh—to understand how various directors have adapted Greek tragedy in twentieth- and twenty-first-century theatre.^2^ Yet one does not need to deconstruct a text, or even to point to the gaps in meaning which persist in any textual encounter, to explore fragmentation, but rather can turn to the presence of actual fragments in the modern world.

Creative engagements with tragic fragments represent a growth area within artistic practice and encompass Timberlake Wertenbaker’s response to Sophocles’ *Tereus*, *The Love of the Nightingale*, Colin Teevan’s reconstruction of Euripides’ *Alcmaeon in Corinth*, and Laura Swift and Russell Bender’s forthcoming engagement with Euripides’ *Cresphontes*.^3^ Analysing this cluster of theatrical productions allows us to think about the postmodern preoccupation with fragments in a lucid way. Yet one recent fragmentary reception is particularly noteworthy, namely Punchdrunk’s 2017 *Kabeiroi*, as it is indicative not only of how we respond to fragmentary material in general, but also of the unique artistic opportunities offered by fragments for realization in immersive theatre. Immersive theatre is a form of interactive theatre where audiences are incorporated into the world of the performance and positioned as roaming participants whose own actions (where they go and which actors they follow) and tactile engagement with the set (which locations they enter, objects they touch) dictate their version of the production’s narrative and its meaning. The aim of most immersive performances is to allow the audience to feel part of an alternate reality, or as Lyn Gardner describes ‘as if they have dropped down a rabbit hole into a parallel universe’.^4^ Often, such immersion is created through elaborately detailed set design; however, in Punchdrunk’s *Kabeiroi* less proved more, and the sense of absence contained within the fragments’ form created a productive impetus for the creation of the unified imaginary world necessary for a ‘deep’ form of immersion.

In this essay, I analyse *Kabeiroi* and the strategies through which Punchdrunk created an immersive experience from the tragic fragments.^5^ On the one hand, I am concerned with documenting the production, as the available traces of performance are themselves fragmentary due to *Kabeiroi*’s limited run and the company’s decision not to issue press tickets.^6^ On the other, I seek to highlight what we can learn from the production about the potential of tragic fragments for the creation of other immersive experiences, by analysing hitherto unappreciated details of the performance regarding the source text and its context. My analysis reveals how a sense of confusion and a lack of resolution is inherent in the dramaturgy of the material. My central claim is that that the sense of yearning for completion prompted by the fragments mirrors what Adam Alston terms the participatory incentive and goal of idealized Experience [sic] in immersive theatre. Alston claims that immersive theatre thrives on ‘producing the belief that there is always more to be experienced and that an experience can always be bettered or that the environment can be explored in other ways, with greater gusto, or with a greater investment of belief and commitment’.^7^ Rather than the sense of confusion contained in the fragmentary form working to the detriment of the performance, in *Kabeiroi* it echoed the audience’s pursuit of Alston’s idealized Experience within the immersive form. I suggest that the synergy between the form of tragic fragments and the form of immersive performance makes for a unique partnership which holds considerable artistic potential.

Punchdrunk’s *Kabeiroi* ran from 26 September to 5 November 2017.^8^ British theatre company Punchdrunk are one of the most influential practitioners of immersive theatre internationally. They are most famous for productions involving masked, roaming audiences being incorporated into the world of the performance, which often take place in multi-storey warehouses for audiences that number into their hundreds per performance.^9^*Kabeiroi* was a radical departure from what, over the past two decades, had become a tried-and-tested format for the company and was instead a return to the innovation and experimentation of the company’s early days.^10^ The production capitalized upon the enigma surrounding Aeschylus’ lost play to create a four-to-six-hour durational adventure for just two audience members at a time. Several pairs experienced *Kabeiroi* on each day of the run, commencing the experience at staggered times to ensure they did not cross paths. Each pair was positioned as the protagonist(s) of the performance and navigated their experience on the streets of London; the company note that attendees were ‘audience-participants’ who were ‘charged with being present, responsive and integral to the development of the work in form and content’.^11^ In practice, this meant that the audience was tasked with completing a quest narrative and was required to follow customized real-time instructions delivered via an audio headset, text messages, hand-delivered notes, and through books and leaflets retrieved from lockers and storage units. Participants were tracked through satellite navigation throughout the performance. For director Felix Barrett, the fundamental purpose of the project was to blur ‘the liminal space between the imagined and everyday worlds joined by the narrative journey’.^12^ Occasional audience interactions with planted actors in the street achieved this goal and made it difficult for spectators to tell who was a performer and who was a member of the public.

Punchdrunk’s *Kabeiroi* is clearly far removed, in form and content, from a Greek tragedy. However, although we can be certain that Aeschylus’ *Kabeiroi* did not involve audience immersion (at least in the Punchdrunk sense), the reality is that we know very little about this non-extant drama, from which only three fragments survive. The first fragment involves a protagonist stating ‘but I do not make you an omen of my journey’ [TrGF 3 F95]. The second involves the chorus promising ‘that there shall never be a dearth of jars, neither of wine nor water, in this wealthy home’ [TrGF 3 F96], while the final fragment sees the chorus, according to Plutarch, ‘playfully threatening’ to make the house run short of vinegar [TrGF 3 F97].^13^ Athenaeus records that the play featured Jason and the Argonauts, and that Aeschylus’ depiction of them in the play included the first instance of drunkenness on the tragic stage [10.428f], while a scholion to *Pythian 4* notes that the drama contained a full catalogue of the Argonauts.^14^ The Kabeiroi deities featured as the play’s chorus, who on the basis of wider mythology we can assume at some point initiated Jason and the Argonauts into their mystery religion.^15^

Despite knowing so little about the plot of *Kabeiroi*, scholars have nevertheless proposed several hypotheses about the play, a selection of which Punchdrunk utilized for their production. Hypotheses about *Kabeiroi* are usually based upon our knowledge of Aeschylus’ other Argonaut plays. The first of these, *Phineus*, was performed in 472 BC along with *Persians, Glaucus of Potniae*, and the satyric *Prometheus*.^16^ Classicists have traditionally grouped the remaining non-extant plays (*Kabeiroi*, *Women* [or Men] *of Lemnos*, *The Argo* [or Oarsmen], and *Hypsipyle*) as a connected tetralogy, and if this proposal is accepted it provides two likely options for *Kabeiroi*’s location, both of which are based upon the chronology of the Argonauts’ voyage.^17^ The first option, favoured by Alan Sommerstein, posits an order of *Women of Lemnos*, *Hypsipyle*, and *Kabeiroi*, followed by the satyr play *The Argo*.^18^ Sommerstein’s proposal gives the three tragedies a loose unity of place, with all occurring on the island of Lemnos and likely telling, in turn: (1) the myths surrounding the Lemnian crime; (2) the aftermath of the crime and the Argonauts’ arrival at Lemnos and sexual intercourse with the Lemnian women; and (3) the Argonauts’ initiation into the cult of the Kabeiroi; with (4) *The Argo* depicting a chorus of satyrs attempting to join the Argonauts on the Argo, and possibly being prevented by the ship herself from doing so.^19^ The second option, proposed by Bernard Deforge, follows the mythological narrative contained in Apollonius of Rhodes’ *Argonautica* [1.915–21]^20^ and sees an order of *The Argo*, *Hypsipyle*, and *Kabeiroi*, with *Women of Lemnos* as the satyr play. Deforge’s preferred order would see the plays in turn likely tell the myths surrounding: (1) the start of the Argonauts’ voyage; (2) their first stop at Lemnos and their sexual intercourse with the Lemnian Women (leading to Queen Hypsipyle bearing Jason two children); and (3) their second stop at Samothrace and their initiation into the cult of the Kabeiroi; while (4) *Women of Lemnos* as satyr drama might mock the foul smell with which Aphrodite cursed the female inhabitants of Lemnos and which would ultimately lead to the women murdering the island’s male inhabitants.^21^ There is no definitive evidence indicating that one grouping is more likely than the other, although Sommerstein’s arrangement may be more likely given that an anthropomorphized Argo seems more suited to Satyr drama, and the Lemnian women a more fitting collective for a tragic chorus rather than a group of satyrs. Either way, Matthew Wright reminds us that the mere idea of an Argonautic tetralogy is based upon the appeal of such a performance, rather than any evidence indicating that the plays were performed together.^22^ Nevertheless, even if the hypotheses do not shed light on *Kabeiroi,* they remind one of the mythic framing of the Argonauts’ journey which would have been part of the original audience’s horizon of expectation.

No other surviving evidence about the cult of the Kabeiroi and the Argonauts’ initiation helps to confirm the location of the tragedy. The Argonauts’ initiation into a mystery religion is only attested in extant literature to have occurred at Samothrace. The specific mystery cult into which they are initiated at Samothrace is unstated in both Apollonius of Rhodes and Valerius Flaccus; however, Herodotus’ [2.51] mentioning of the Samothracians as celebrating the Mysteries of the Kabeiroi has led to an assumption that it was the cult of the Kabeiroi into which the Argonauts were initiated.^23^ Yet both Lemnos and Samothrace contain archaeological evidence indicating the presence of the Kabeiroi cult, and Walter Burkert assumes that Aeschylus’ tragedy would have been set in Lemnos on the basis of excavated physical evidence.^24^ The tangential evidence supporting the Lemnian location is in the form of wine vessels, which constitute the most substantial archaeological find at the Lemnian Kabeiroi sanctuary.^25^ These material finds are linked to the theme of intoxication that was present in *Kabeiroi*, evinced in Athenaeus’ comment regarding staged drunkenness and the surviving fragment in which the chorus promise never to make a home empty of wine.

The hypothesis that Aeschylus’ *Kabeiroi* involved the Argonauts being initiated into the cult of the Kabeiroi at Lemnos is built upon shaky ground. However, the available evidence indicates that this scenario is at least as likely as any other scenario. If it was the case, *Kabeiroi* would have provided an intriguing alternative to the narrative set out in the later Argonautica epics. Either way, the Lemnian setting for the Argonauts’ initiation is the frame that Punchdrunk settled upon for their production. It is reflected in Punchdrunk’s use of an all-female cast to reflect the all-female island of Lemnos. Audience members also received a card debossed with a capitalized Greek lambda after the performance to indicate that they were now card-carrying initiands of the Lemnian cult. Irrespective of whether Punchdrunk’s interpretation of the fragments corresponds to Aeschylus’ original play, their process of interpretation is illuminating as it reveals the inevitable sense of lack to be found in any engagement with fragments. In any encounter with non-extant tragedy, artists are faced with a choice between preserving the sense of mystery and lacunae or moving towards a hypothetical reconstruction which might aspire towards a sense of completeness. Irrespective of the artist’s approach, the outcome will have an undercurrent of absence. Rather than perceive the inability to reconstruct Aeschylus’ plot in any further depth as a limitation, Punchdrunk saw the lacunae as a tantalizing opportunity and from the text’s skeletal surviving basis created *Kabeiroi* anew.

Punchdrunk’s *Kabeiroi* did not seek to reconstruct Aeschylus’ lost tragedy, but rather was a new and innovative performance which preserved the sense of lacunae contained in the surviving material by not featuring a unified overarching narrative. Only one of the three surviving fragments was explicitly engaged with, and even this engagement, with the line ‘but I do not treat you as an omen of my journey’, was done in the publicity for the performance rather than the show’s content.^26^ The production’s form, however, had a tripartite structure which paralleled the number of surviving fragments. These three sections took the form of an audio tour, a scavenger-hunt-style adventure done as a pair, and then one done as an individual. The audio tour led audiences through Bloomsbury and into the British Museum. All Punchdrunk productions employ what the company refer to as a ‘cross-fade’, which is a mechanism ‘to enable immersion and encourage the audience-participant to cross, physically and imaginatively, from the everyday world into the world of the event’.^27^ The audio tour provided the cross-fade in *Kabeiroi*; noise-cancelling headphones blocked out the sounds of the wider world, while the voiceover encouraged audience-participants to concentrate upon certain elements of their surroundings, for example to imagine the secret gatherings of the Bloomsbury group, to look up and see gargoyles peering down from the city’s architecture, and to try to make eye contact with those passing by. At the British Museum, after being directed to gaze at a series of specified objects, audience members were stripped of their headphones and handed a folded card with a wax seal bearing instruction to open at the main entrance of the Museum.

The second part of the performance commenced on the steps of the Museum. The card asked spectators to indicate their acceptance of their ‘chosen’ status by raising their hand.^28^ A scavenger-hunt-style experience then followed under the pretence of preparing the participants for a journey, with text messages instructing the pair to retrieve multiple objects (swipe cards, a bag from a YMCA locker which contained added details regarding the source material via an annotated and scrapbooked copy of Robert Graves’ *The Greek Myths*, and a votive from a storage facility). As night fell planted performers emerged at various locations, for example to chase the pair out of the storage facility, which helped create the illusion that audience members were at the forefront of an epic quest. Text messages reinforced this notion, encouraging the audience away from those who might obstruct their path. Ultimately, each pair was directed to the Grange Langham hotel to meet with a guide, at which point the two audience members became separated.

The final third of the performance took place after dark and was the section of *Kabeiroi* that was most closely affiliated with the company’s interpretation of the Aeschylean material. Armed with a map and a mobile phone, each individual separately navigated the streets of London in search of various clues and phone numbers, which when dialled involved pre-recorded messages which addressed the spectator as ‘Jason’ and referred to a storm closing in and the desperate need for the individual to reach safety in advance.^29^ After navigating (separately, via the London Underground) to a warehouse in Tottenham Hale, each audience-participant experienced a more traditionally immersive dénouement involving a designed performance space filled with actors. One audience-participant was instructed to attempt, Pentheus-style, to infiltrate a mysterious cult, only to be discovered by an actor, and then rescued by the other audience-participant. Both audience members were then required to escape from the actors, make their way to an altar, perform a libation, and ultimately undergo an initiation of sorts.

Punchdrunk’s response to the ancient material was experientially powerful, using the city of London as a stage and at various moments casting the audience member as the performance’s protagonist. The spectator was active throughout but given limited agency to influence or change the structure of the performance, in much the same way that a tragic protagonist like Oedipus is unable to deviate from their predetermined fate.^30^ In a reflection of the content of the surviving fragments, the narrative of Punchdrunk’s reception remained somewhat opaque throughout. The opacity was particularly evident in the connections between the three sections, which formally elevated the stakes surrounding the audience-participant’s involvement in the performance but did not develop the plot linearly. The performance’s form represents the company’s attitude towards responding to fragments. Punchdrunk did not seek to create a unified, linear reconstruction of *Kabeiroi*, but nor did they overtly represent Aeschylus’ fragments in performance. Rather, the production’s form preserved a sense of lacunae, rather than the actual lacunae, and used the conceit of responding to an unknowable past to frame the audience’s conceptual engagement with the performance. As such, the audience’s familiarity with Aeschylus’ non-extant tragedy, which is a relatively obscure play even to many classicists, became less significant than the audience’s awareness that the performance was offering engagement with a lost source from antiquity to which they would need to contribute imaginatively.

Punchdrunk’s preservation of a sense of fragmentation makes clear how a sense of yearning and incompleteness is built into responses to fragmentary tragedies. In other words, Punchdrunk’s emphasis upon the form of ancient fragments, rather than their content, indicates that it is ancient fragments in general, rather than the fragments of *Kabeiroi* specifically, that offer artistic potential for immersive experiences. When a sense of absence is accepted as a precondition of performance, then the theatrical possibilities of fragments within immersive theatre become apparent. Joshua Billings’ concept of the erotics of reception, which is sensitive to how the classical world is both present and absent in modernity and ‘establishes a dialectic of lack and resource that leads to a productive relation to antiquity’, provides a useful way of articulating the productiveness of this sense of absence.^31^ Billings’ argument is based upon an analysis of Hölderlin’s *Hyperion* as a reception of Plato’s *Symposium* and the dialectic it creates between the presence of desire and the absence of its fulfilment; Billings argues that this dynamic is representative of all processes of classical reception, where the absence and untimeliness of antiquity can be a genuinely productive force which is ‘conditioned by a desire that makes the relation to the ancient past simultaneously an imperative and an impossibility’.^32^ On the one hand, Punchdrunk’s *Kabeiroi* offers a literal parallel to Billings’ argument, where we can chart our relationship between the actual fragments and our desire for the lack of the rest of the play. On the other, however, the production’s undercurrent of an unfulfillable desire for antiquity shows the conceptual relevance of Billings’ ideas, too, particularly as the absent nature of the ancient world in *Kabeiroi* involves not just the textual remnants of Aeschylus’ play but also the initiation into a mystery religion that it depicted. Desire for an unknowable past drove audience immersion in Punchdrunk’s *Kabeiroi*.

Two moments of *Kabeiroi* highlight how the sense of yearning associated with fragments can prompt a deep form of audience immersion, namely the transition from the first section of the performance into the second, and the finale of the experience. To be clear, when I posit that the use of fragments in *Kabeiroi* facilitates a deep form of immersion I mean that the sense of lacunae allows for the audience to feel that they are in a unified imaginary world, through the content that they are required to contribute mentally to the performance to fill in any perceived lack. The sense of immersion is deep because it mirrors, or doubles, what Alston identifies as the drive towards an ever-elusive, more perfect, idealized Experience that underpins audience engagement in immersive theatre.^33^ Although the process of projecting material onto a text to create interpretation occurs within any textual encounter, the process of filling in the gaps of meaning in a fragmentary text is to a different extreme and offers a different type of interpretative freedom, which mimics an audience’s experience in immersive performance. In immersive theatre, each audience member’s experience is unique to a different extreme than is the case in other theatrical performances, due to the potential for spectators to see entirely different interactions and have personalized encounters. No two individuals have identical experiences, and the value and meaning to be found in each moment of performance is subjective and is shaped by an audience member’s choices regarding, for example, where to navigate to or how to respond to a prompt or invitation.^34^ The fact that *Kabeiroi* would last for anywhere between four and six hours depending on the audience’s engagement with and choices within the performance testifies to this range of experiences. There is no such thing as an objectively complete narrative or experience within immersive performance, but simply unlimited partial, personal experiences. It is of course possible for the sense of yearning to be perceived as unfulfilling. Critic Andrzej Lukowski, for example, argues that *Kabeiroi* was ‘somewhat let down by a plot that a decent dramaturg could fix without much bother. It’s about some Greek gods offering us… immortality. I think. And some other Greek gods… trying to stop us? Maybe?’.^35^ However, when the sense of partiality is accepted as a precondition of the performance, then the interpretive freedom created can foster a depth of immersion. Such depth was particularly available in *Kabeiroi* as the participatory strategies required by the performance’s form mimicked the cognitive strategies required to process a narrative framed as being fragmentary.

The first moment that illuminated this dynamic occurred at the end of the first section of the performance, when the audio tour led each pair of audience-participants to the British Museum. Until this moment, the performance had been entirely detached from the Greek source text. Pre-show publicity indicated that Aeschylus’ lost *Kabeiroi* would be re-imagined in Punchdrunk’s reworking, and specifically ‘the ancient Greek myth of the women of Lemnos’, but until the British Museum the audio tour had focussed not on the presence of the classics in London but either on the Bloomsbury literary group or on Bloomsbury’s sculptures.^36^ At the Museum’s entrance, the voiceover instructed the audience-participants that they were arriving ‘at where it all began’ and that they would be going right ‘to the beginning, to ancient Greece’.^37^ The voiceover then directed the audience to walk from Room 6 (early Greece) to Room 17 (the Nereid monument) and instructed each individual to gaze only on specific objects, cautioning ‘don’t get distracted by other treasures—they are not why we are here’. The objects that the audience-participants were directed to gaze upon included a vase for drinking wine with a procession of gods around the rim, a cluster of seven Mycenaean terracotta female figures with their arms outstretched, and a 6.35 cm bronze bell excavated from the Theban Kabeiroi sanctuary.^38^ Due to the paratextual information released through the pre-show publicity, the audience experience throughout the cross-fade was in part arguably one of waiting for evidence of the play’s connection to the source text. At this eventual first reference, occurring close to an hour in, the music through the audio headset began to swell. Spectators were then directed, while listening to the increasingly rich soundscape, to the Nereid monument, which is a funerary monument featuring three full-size headless Nereids in wind-blown drapery positioned between the tomb’s columns. The transition from a small, unassuming Kabeiroi bell to the imposing female sculptures had the effect of equating the Kabeiroi cult with powerful female figures, and this sense was further emphasized through the sudden appearance of a female actor who came and stood between the monument and the pair of spectators, fixing them with an intense gaze. Slowly, she took each audience-participant’s hand. The three people shared a silent moment, oblivious to the other museum attendees who continued to bustle around, before the performer released the two hands, gave the pair their card of instructions, and disappeared.

The efficacy of the transition between the first and second parts of the performance lay in the framing of the moment as a response to a fragmentary tragic source and the staging of the reimagining of the source in a public setting which embodied a heightened connection to antiquity. Although all Punchdrunk work can be described as immersive, *Kabeiroi* differed to the highly controlled and designed environments of Punchdrunk’s masked performances. In masked productions, Punchdrunk use a canonical source text to flesh out the world of the performance. Barrett describes his process for masked performances as ‘like a logical puzzle in itself. You dissect it [the source text] and then think, this little couplet here will look beautiful in a cupboard, this passage here is obviously core narrative, this bit here is describing atmosphere so that’s in the lighting’.^39^ That Punchdrunk usually choose to ‘fragment’ canonical texts reveals the power of removing linearity for creating immersion. James Frieze refers to the process of the audience reconstructing canonical stories in Punchdrunk masked shows as a form of ‘textual archaeology’.^40^ Although there is power in the agency that engaging in ‘textual archaeology’ provides, the process can cause frustration if the immersant struggles to find and piece together the expected, known story. One could create a durational, promenade performance using any source text, or none at all, but when *Kabeiroi* is compared to Punchdrunk’s other work it is arguable that the more fragmentary source text and unknown narrative inspired a particularly deep form of immersion as an end result.^41^ The fragments could not facilitate a unified imaginative world with the level of detail contained in Punchdrunk’s masked work but could only operate by coexisting with everyday reality and ensuring, as Barrett argues, that the audience ‘feel life becoming a work of art’.^42^ The avoidance of references to the tragic fragments until an hour into the experience underlined the futility of using the ancient content to build, by itself, an imaginary world and stressed to the audience that the world in which they were being immersed was one of their own creation.

The benefits found in creating immersion through fragments were furthered due to the performance taking place in an outdoor setting rather than a designed performance space. In his exploration of immersive experiences which take place in the familiar environment of the cityscape, Alston argues that the sense of immersivity relies upon a ‘productive investment’ of the audience and involves meaning being made through the ‘tension between the performing city and the urban dramaturgy of an immersive theatre performance that a participating audience member is invited to negotiate’.^43^ Aeschylus’ fragmentary tragedy heightened this tension in *Kabeiroi* as it embodies a dialectic of lack. Consequently, as the negotiation between the dramaturgy of the city and the dramaturgy of the performance is made more complex, the audience’s investment in the world of the performance is heightened. The spectator is not detached from the immersive experience by a mobile fourth wall in the form of a mask, and any accusations of intellectual passivity or stultification become negated due to the productivity of the audience’s negotiation between the experience and the fragmentary source and their protagonist position and the environment.^44^ By holding back references to the text until the transition from the first to the second section of the performance, any expectation of seeing a reconstruction of Aeschylus’ *Kabeiroi* was shattered. When the mythic context was finally tangentially referenced, it was done in a way which made clear that the performative world would be overlaid and coexist with everyday reality.

The finale of *Kabeiroi* provides an additional example of the efficacy of using ancient fragments to create immersive theatrical experiences. Barrett refers to the finale of any Punchdrunk production as a ‘crescendo’ and argues that ‘It is a vital element of any Punchdrunk experience, often being part of the early concept for a production … when creating any project we always work back from its emotional peak, the zenith of the crescendo. Knowing what that will be early on enables us to form the show’.^45^ Barrett’s original vision for *Kabeiroi*’s finale involved audiences journeying from central London to Beckenham in a city-to-country pilgrimage which echoed the journey from Athens to Eleusis in the more famous Eleusinian mysteries.^46^ Although for Barrett the loss of the Beckenham venue appears a source of disappointment, audience comments indicate that the substituted finale remained an arresting experience, with Michael Smith describing it (somewhat sensationally) as ‘life-changing’ and Alice Saville as ‘a stunningly lit, blisteringly beautiful encounter with the gods’.^47^ Without the fragmentary presence of Aeschylus’ play underpinning the experience perhaps the crafting of *Kabeiroi* to exist only within the cityscape palette would have felt unsatisfying. However, the traces of the tragic fragments compensated for the missing city-to-country pilgrimage by operating as a conceptual frame for audience-participants to pin their experience upon and by adding a mythic subtext.

The final moments of *Kabeiroi*, after the pair of audience members had been reunited, involved the audience-participants running to a candlelit shrine in a dark room, where they were instructed to place the votive sourced from the storage facility earlier in the performance and then to make a libation at the foot of a stone bust of a female figure. The audience was then instructed to back away into a final space and slowly turn around, where two female performers were waiting with arms outstretched. They embraced the two audience members, before a third performer appeared, who was the woman from the British Museum.^48^ She smiled at the audience and caressed their faces. A warm, blindingly bright light then filled the space. When it dimmed and the audience could once again see the space it became apparent that the performers had vanished. Instead, a door in the far-right corner was now open, inviting audiences back into the real world.

The simple finale sequence lasted only a few minutes but nevertheless succeeded in highlighting the company’s chosen interpretation of the narrative of Aeschylus’ tragedy, namely the welcoming of Jason and the Argonauts after a perilous journey into the all-female island of Lemnos and their initiation into the cult of the Kabeiroi.^49^ Like all ancient mystery religions little is known about the process of initiation into the cult of the Kabeiroi. The details that are known were not restaged in *Kabeiroi*, and indeed not even the known stages of initiation into the more famous Eleusinian mysteries were staged beyond the process of going from darkness to a blinding light.^50^ Yet just as in the British Museum *Kabeiroi* drew upon the fragmentary nature of the source text to invite the audience to experience the fragmentary material as a palimpsest underlying the modern world, in the finale traces of the Aeschylean play were once again inserted as the force underpinning the experience. By collapsing the audience’s role with that of the tragedy’s protagonist, Punchdrunk had set the scene for the finale to also collapse reality with ritual and feature audience initiation into the cult of the Kabeiroi. As both the tragedy and the initiation rituals were unknown quantities, Punchdrunk could immerse audiences in a way which did not involve ‘fact checking’ and risk pulling them out of the experience, but instead invited spectators to accept the company’s artistic licence and to suspend their disbelief. The finale invited the audience to experience the content of a lost initiation ritual from an unknowable past, which reflected the elusive form of an idealized immersive Experience. Bastien Goursaud and Déborah Prudhon have argued that Aeschylus’ text similarly blurred the boundaries between ritual and reality, as the cult into which Jason and the Argonauts were initiated was a real mystery religion.^51^ Punchdrunk’s modern reworking, they posit, is thus a ‘symmetrical image’ of Aeschylus’ play as it did not just reinvent the initiation but ‘made us perform it’.^52^ Punchdrunk’s *Kabeiroi*, they argue, thus had ‘transformative value’ in that it encouraged ‘us to see differently, to instil a difference in the way we perceived urban life and the familiar streets of Bloomsbury and central London’.^53^ Although some audience members may have found the modern reception to hold transformative value, it seems something of a reach to imply that Aeschylus’ play collapsed fiction and reality simply through alluding to a real religious ritual.^54^ However, the fact that the two plays were not necessarily equivalents is not detrimental to Punchdrunk’s finale, which still mined the power of collapsing ritual and reality to facilitate audience immersion, and which built upon the already productive sense of untimeliness established through the company's engagement with tragic fragments.

There is no one way of achieving audience immersion in contemporary theatre, and indeed experimenting with tragic fragments as a source text is far from a fool-proof method. However, Punchdrunk’s *Kabeiroi* demonstrates that when the simultaneous presence and absence of the source material is accepted as a precondition of a performance, rather than a dramaturgical flaw, then there are considerable artistic benefits to the approach. In *Kabeiroi* the tragic source layered the audience experience and gave complexity to the performance’s content while at the same time resisting the impression that it alone could create a unified imaginary world or a historical reconstruction of a religious experience. As such, the performance could only function by coexisting with everyday life. Rather than acting as a barrier to immersion, the collapsing of ritual and reality and the empowerment of the audience to fill in the lacunae fostered a deep and organic form of immersion by allowing each individual to position themselves within a unified imaginary world of their own creation. The aspiration towards an external sense of completeness was, in *Kabeiroi*, rendered moot, and instead each conceptual imaginary world was as unique as each audience-participant’s subjective experience within the performance. The ever-present sense of yearning for completion contained in Punchdrunk’s response to tragic fragments mirrored what Alston identifies as the audience’s pursuit of an unattainable, idealized Experience in immersive performance. The synergy between the form of the source text and the theatrical form of immersive performance made for a unique partnership which fostered a deep form of immersion. Given today’s preoccupation with fragmentation, Punchdrunk’s strategy in *Kabeiroi* represents a fertile method for contemporary artists. The innumerable other surviving fragmentary texts hold untapped potential for an entirely new direction of classical performance reception.

## Footnotes

1 E. Ioannidou, *Greek Fragments in Postmodern Frames: Rewriting Tragedy 1970–2005*, Oxford, 2017.

2 E. Fischer-Lichte, *Dionysus Resurrected: Performances of Euripides’* The Bacchae *in a Globalizing World*, West Sussex, 2014, pp. 72*–*89. Justine McConnell also uses the term sparagmos to illuminate creative artists’ engagement with antiquity. See J. McConnell, ‘Postcolonial *Sparagmos*: Toni Morrison’s *Sula* and Wole Soyinka’s *The Bacchae of Euripides: A Communion Rite*’, *Classical Receptions Journal*, 8, 2, 2016, pp. 133*–*154.

3 See T. Wertenbaker, *The Love of the Nightingale; and, The Grace of Mary Traverse*, London, 1989, C. Teevan, *Alcmaeon in Corinth: after a fragment of Euripides: First Performed as ‘Cock of the North’*, London, 2017, and Potential Difference, *Fragments*, accessed 17 February 2020, <https://www.potentialdifference.org.uk/productions/fragments>. Swift and Bender’s *Fragments* was due to be staged in 2020 but was delayed due to COVID-19.

4 L. Gardner, ‘Is Immersive Theatre Growing Up or Growing Too Big, Too Quickly?’, *The Stage*, 2014, accessed 23 July 2020, <https://www.thestage.co.uk/features/is-immersive-theatre-growing-up-or-growing-too-big-too-quickly>.

5 I was the academic consultant on *Kabeiroi*. I was involved in the performance’s research and development period but did not make creative decisions, and nor did I know the production’s final form before experiencing it.

6 Only 864 tickets were available for *Kabeiroi*, and demand far outstripped supply. The difficulty of obtaining tickets for *Kabeiroi* was discussed extensively in the media. See A. Needham, ‘Punchdrunk’s luxury rebrand is the theatrical version of gentrification’, *The Guardian*, 28 September 2017, accessed 17 February 2020, <https://www.theguardian.com/stage/2017/sep/28/punchdrunk-kabeiroi-luxury-brand-theatrical-gentrification>, and A. Lukowski, ‘Theatre ballots are a dick move that shut out dedicated fans’, *The Stage*, 2017, accessed 22 January 2020, <https://www.thestage.co.uk/opinion/2017/andrzej-lukowski-theatre-ballots-dick-move-shut-dedicated-fans/>. Journalists were invited to enter the ballot for the right to buy tickets. A small number of reviews exist from those who were successful in the ballot.

7 A. Alston, ‘The Promise of Experience: Immersive Theatre in the Experience Economy’, in *Reframing Immersive Theatre: The Politics and Pragmatics of Participatory Performance*, ed. J. Frieze, London, 2016, 243–264 (247).

8 Punchdrunk, ‘Kabeiroi’, *Punchdrunk*, 2020, accessed 19 February 2020, <https://www.punchdrunk.org.uk/project/kabeiroi/>.

9 On the history of immersive theatre as a concept within artistic and academic circles, see J. Machon, *Immersive Theatres: Intimacy and Immediacy in Contemporary Performance*, London, 2013, pp. 65*–*66.

10 James Temperton, for example, writes of Punchdrunk’s work that ‘what was once theatrical innovation is in need of a rethink […] And so, having broken down the fourth wall, [director Felix] Barrett is now trying to rip up the foundations’. See J. Temperton, ‘Punchdrunk’s next great act’, *Wired*, 2017, accessed 4 October 2019, <https://www.wired.co.uk/article/punchdrunk-kabeiroi-felix-barrett-interview-sleep-no-more-2018>. On the company’s early days, see J. Machon and Punchdrunk, *The Punchdrunk Encyclopaedia*, London and New York, 2019, pp. 3*–*8. The publicity for the production noted ‘*Kabeiroi* is different to what’s come before’. See Punchdrunk, ‘Kabeiroi’ (n. 8 above).

11 Machon and Punchdrunk, *The Punchdrunk Encyclopaedia* (n. 10 above), p. 22, and on the audience as character in *Kabeiroi* see p. 232. Sophie Nield has written critically about the phenomenon of positioning audiences as characters in immersive theatre, and the awkwardness and embarrassment that can arise. Her criticisms, however, are less relevant to *Kabeiroi*, as in *Kabeiroi* the audience had greater agency and were not usually aware as to when they were interacting with (and being observed by) performers. See S. Nield, ‘The Rise of the Character named Spectator’, *Contemporary Theatre Review*, 18, 4, pp. 531*–*544.

12 Machon and Punchdrunk, *The Punchdrunk Encyclopaedia* (n. 10 above), p. 156.

13 A. Sommerstein, *Aeschylus: Fragments*, Cambridge, Massachusetts, and London, 2009, p. 109.

14 See ∑ Pindar, *Pythian* 4.303b.

15 The Argonauts are initiated into the cult of the Kabeiroi in both Apollonius Rhodius [1.915*–*21] and Valerius Flaccus [2.432*–*42].

16 TrGF 3 T55a. See also M. Wright, *The Lost Plays of Greek Tragedy (Volume 2): Aeschylus, Sophocles and Euripides*, London, 2018, p. 13.

17 *Kabeiroi* has previously been posited as satyric because of Athenaeus’ note about the play staging drunkenness; however, given that Ath. specifies that *Kabeiroi* was the first example of drunk characters being brought onto the *tragic* stage, the proposal is unconvincing.

18 Sommerstein, *Aeschylus* (n. 13 above), pp. 126*–*7. The *Women of Lemnos–Hypsipyle–Kabeiroi* order is also preferred by Gantz. See T. Gantz, *Early Greek Myth: A Guide to Literary and Artistic Sources*, Baltimore and London, 1993, p. 346, and for further detail on the potential plot of *Lemnian Women* and *Hypsipyle* see pp. 345*–*6.

19 Sommerstein, *Aeschylus* (n. 13 above), pp. 14*–*15. Sommerstein later proposes that *Hypsipyle* and *Kabeiroi* may have been performed in the alternate order, with *Hypsipyle* ‘maybe centring on Jason’s discovery of the Lemnian women’s crime and his abandonment of Hypsipyle and departure from the island’. Such an order opens up the possibilities for the content of *Kabeiroi*, enabling it to feature the Argonauts’ arrival on Lemnos, their swearing of an oath to have intercourse with the women upon disembarking, their reproduction with the women, and their initiation into the mystery religion; however, it does not affect the possible Lemnian setting of the play. See A. Sommerstein, *Aeschylean Tragedy*, London, 2010, p. 37.

20 Also Val. Flacc. 2.432*–*42.

21 B. Deforge, ‘Eschyle et la légende des Argonautes’, *Revue des Études Grecques*, 100, 1987, pp. 30*–*44, esp. p. 39, 43.

22 Wright notes that ‘the titles and fragments are unhelpful in this respect [i.e. in indicating a connected tetralogy], since they tell us almost nothing and could be arranged in any order’. See Wright, *The Lost Plays* (n. 16 above), p. 21.

23 For an assessment of mystery religion at Samothrace see J. Bremmer, *Initiation into the Mysteries of the Ancient World*, Berlin, 2014, pp. 21*–*54.

24 W. Burkert, *Greek Religion*, Oxford, 1985, p. 281. The Kabeiroi were also worshipped at Thebes, although there is no evidence indicating that Aeschylus’ tragedy was set there. On the Theban Kabeiroi see R. Fowler, *Early Greek Mythography (Volume 2): Commentary*, Oxford, 2013, p. 38 and W. Burkert, *Greek Religion*, Oxford, 1985, pp. 281*–*282.

25 Fowler, *Early Greek Mythography* (n. 24 above), p. 42 and Burkert, *Greek Religion* (n. 23 above), p. 281.

26 Punchdrunk, ‘Kabeiroi’ (n. 8 above). The publicity was carried out through Punchdrunk’s website and a press release.

27 Machon and Punchdrunk, *The Punchdrunk Encyclopaedia*, (n. 10 above), p. 69.

28 The card read ‘You have both been chosen, now together you must decide. Your journey ahead may be filled with peril but the rewards will be plentiful. If you are ready and willing to accept then each raise your hand now’.

29 Bastien Goursaud and Déborah Prudhon transcribed one of the voicemail recordings: ‘It’s gaining on you. You must find shelter before the storm hits. You’re running out of time, Jason. Go now, into King’s Cross. Find somewhere safe and brace yourself against the wind. Your guide must know when you’re safe. Only she can help you now. You must send her a message. Remember, eyes are everywhere’. See B. Goursaud and D. Prudhon, ‘*Kabeiroi* by Punchdrunk’, *Études britanniques contemporaines* [En ligne], 54, 2018, accessed 8 October 2019, <https://journals.openedition.org/ebc/4408>. Both individual audience*-*participants were addressed as Jason, although as they were now experiencing the production individually neither would be aware of this at the time.

30 I address the wider debates surrounding audience agency within Punchdrunk’s *Kabeiroi* and the relationship between this form of immersion and Jacques Rancière’s notion of the emancipated spectator in E. Cole, *Postdramatic Tragedies*, Oxford, 2019, pp. 212*–*213.

31 J. Billings, ‘Hyperion’s symposium: an erotics of reception’, *Classical Receptions Journal*, 2, 1, 2010, pp. 4*–*24 (22).

32 Billings, ‘Hyperion’s symposium’ (n. 31 above), pp. 21*–*22.

33 Alston, ‘The Promise of Experience’ (n. 7 above). In contrast, I am not following a taxonomic scale of immersivity such as that championed by Machon, who argues that the extent to which specific components (in-its-own world, space, scenography, sound, duration/al, interdisciplinary/hybrid practice, bodies, audience, a ‘contract for participation’, intention, and expertise) are identifiable in a work allows one to gauge how immersive it is. See Machon, *Immersive Theatres* (n. 9 above), pp. 93*–*102.

34 Alston has criticized these participatory processes as a form of entrepreneurial participation which shares values with neoliberalism. See A. Alston, ‘Audience Participation and Neoliberal Value: Risk, Agency and Responsibility in Immersive Theatre’, *Performance Research: A Journal of the Performing Arts*, 18, 2, 2013, 128*–*38 (128).

35 A. Lukowski, ‘The verdict on Punchdrunk’s low-key show Kabeiroi’, *The Stage*, 2017, accessed 22 January 2020, <https://www.thestage.co.uk/opinion/2017/the-verdict-on-punchdrunks-low-key-show-kabeiroi/>.

36 On the pre-show marketing copy see Punchdrunk, ‘Kabeiroi’ (n. 8 above), and on the two audio-tour options see Machon and Punchdrunk, *The Punchdrunk Encyclopaedia* (n. 10 above), p. 156.

37 The entrance that Punchdrunk used was in fact the Museum’s back entrance, which allowed the audience to enter the museum away from crowds and queues and experience the performance in an uninterrupted fashion. Punchdrunk’s decision to frame the British Museum as holding the key ‘to where it all began’, and for ancient Greece to be positioned as ‘the beginning’ buys into an interpretation of antiquity at odds with current discourse surrounding the decolonisation of ancient world studies.

38 The bell dates from 450–400BC and is museum number 1893,1221.1. The bell was inscribed with a series of numbers (93 12*–*21 1). Punchdrunk used these numbers on a poster which was stuck on a building in Tottenham Hale and helped further immerse audiences within the world of the performance by advertising a fictitious music event involving the DJs ‘Argo’ ‘Storm’ and ‘Frenzy’, with MCs ‘Rescue Crew’. The numbers were also used as a password later in the performance.

39 Barrett, quoted in J. Machon, *(Syn)aesthetics: Redefining Visceral Performance*, London, 2009, p. 96.

40 See J. Frieze, ‘Reframing Immersive Theatre: The Politics and Pragmatics of Participatory Performance’, in *Reframing Immersive Theatre: The Politics and Pragmatics of Participatory Performance*, ed. J. Frieze, London, 2016, 1*–*25 (16), and on the relationship between fragmentation and frustration see D. Prudhon, ‘Punchdrunk’s Immersive Theatre: From the End to the Edge’, *Sillages critiques* 24, accessed 28 October 2019. <https://journals.openedition.org/sillagescritiques/6341>.

41 On the idea of Punchdrunk’s *Kabeiroi* exploring ‘deeper immersion’, see Machon and Punchdrunk, *The Punchdrunk Encyclopaedia* (n. 10 above), p. 12.

42 Barrett, quoted in Machon and Punchdrunk, *The Punchdrunk Encyclopaedia* (n. 10 above), p. 232.

43 A. Alston, ‘Making Mistakes in Immersive Theatre: Spectatorship and Errant Immersion’, *Journal of Contemporary Drama in English*, 4, 1, 2016, pp. 61*–*73 (70).

44 On the mask as a form of the fourth wall, see C. Gordon, ‘Touching the Spectator: Intimacy, Immersion, and the Theatre of the Velvet Rope’, *Borrowers and Lenders: The Journal of Shakespeare and Appropriation*, 2013, accessed 22 January 2020, <http://www.borrowers.uga.edu/472/show>.

45 See Barrett, quoted in Machon and Punchdrunk, *The Punchdrunk Encyclopaedia* (n. 10 above), p. 69.

46 On the original planned dénouement for *Kabeiroi*, see Machon and Punchdrunk, *The Punchdrunk Encyclopaedia* (n. 10 above), p. 159.

47 On the finale see M. Smith, ‘Kabeiroi – Punchdrunk’, *Cogdesign*, 2017, accessed 22 January 2020, <https://cogdesign.com/journal/punchdrunk_kabeiroi/>, and A. Saville, ‘Kabeiroi – A Post-Mortem’, *Exeunt Magazine*, 2017, accessed 22 January 2020, <http://exeuntmagazine.com/features/kabeiroi-post-mortem/>. Barrett describes the original finale as follows: ‘You begin in Central London and then you end up in the countryside, from the urban to the rural with a loss of control that comes with that sense of expectation, and once at that site, only then would you all come together as a group of twenty-four [i.e. all twelve pairs who had started the experience that day], all wearing a disguise not knowing who was a performer and who wasn’t, nor who your partner was, with half an hour of a Punchdrunk event in this country-house and its crumbling surroundings which nature has engulfed’. See Barrett, quoted in Machon and Punchdrunk, *The Punchdrunk Encyclopaedia* (n. 10 above), p. 156.

48 The show credits reveal that this performer was explicitly cast as Hypsipyle.

49 On the relationship between the myth of the Kabeiroi and Aesch.’ tragedy see W. Burkert, ‘Jason, Hypsipyle, and New Fire at Lemnos: A Study in Myth and Ritual’, *The Classical Quarterly*, 20, 1, 1970, pp. 1*–*16 (9*–*10).

50 On the known stages of the initiation ritual at Samothrace, including the priest asking the initiand about the worst deed they have committed, the wearing of a purple sash, and the wearing of an iron ring, see W. Burkert, *Greek Religion* (n. 24 above), p. 283. On the process of going from darkness to seeing a great light in the initiation to the Eleusinian mysteries see ibid, p. 288.

51 Goursaud and Prudhon, ‘*Kabeiroi* by Punchdrunk’ (n. 29 above).

52 Ibid.

53 Ibid.

54 Aristotle records in *Nicomachean Ethics* that Aesch. inadvertently revealed secrets regarding the Eleusinian Mysteries [1111a8*–*10]. An ancient commentator on the text further specifies that he was prosecuted for doing so in five non-extant tragedies; however, *Kabeiroi* was not one of the plays in question and there is no evidence that the Argonauts’ initiation was staged in the play. The tragedies are *Female Archers*, *Priestesses*, *Sisyphus the Stone-Roller*, *Iphigenia*, and *Oedipus*. See *Commentaria in Aristotelem Graeca* XX 145.23*–*32.

